# Comment on: Sexual harassment, sexual assault and rape by colleagues in the surgical workforce, and how women and men are living different realities: observational study using NHS population-derived weights

**DOI:** 10.1093/bjs/znad442

**Published:** 2024-01-19

**Authors:** Nithya Krishnan, Ala Szczepura, Jyoti Baharani, J E Tuttle, Lorna O’Doherty

**Affiliations:** Institute of Cardio-Metabolic Medicine, University Hospitals Coventry and Warwickshire NHS Trust, Coventry, UK; Centre for Healthcare and Communities, Institute for Health & Wellbeing, Coventry University, Coventry, UK; Centre for Healthcare and Communities, Institute for Health & Wellbeing, Coventry University, Coventry, UK; Renal Unit, Birmingham Heartlands Hospital, Birmingham, UK; Professor Emeritus, Transplant Surgery, Surgical Immunology and Critical Care, East Carolina Health, Greenville, North Carolina, USA; Centre for Healthcare and Communities, Institute for Health & Wellbeing, Coventry University, Coventry, UK

*Dear Editor*

The article by Begeny *et al*.^1^ reporting the experiences of National Health Service (NHS) surgical staff is of interest. The study adds to growing evidence of issues affecting women in medical professions. These include: gender gaps in residencies; unequal academic progression; pay inequity; and the negative influence of gender stereotypes on medical careers. The authors have shown that use of language and choice of words display elements of covert engendered views in male-dominated specialties; an underlying culture that exposes women to a ‘glass cliff’ in their leadership positions after they have broken through the glass ceiling.

Begeny *et al*.^1^ uncover a culture of sexual misconduct over several years, ostensibly not dealt with by responsible organizations. Underpinning this culture and harm is a continued gender power imbalance with outdated models of surgical leadership in a specialty that is dependent on clinical teams working effectively together. It is remarkable, however, that Begeny *et al*.^1^ make no reference to the ethnicity or the cultural backgrounds of the participants. It appears no such data were collected/reported and no comment on the associated limitations of the research is proffered. Yet, to fully appreciate patterns of sexual violence and abuse, intersectionality is important to consider. Women from diverse backgrounds, could face other abuses, including bullying, microaggression, and disparagement, in addition to sexual violence. This would make them more vulnerable and therefore less likely to report abuse. How different characteristics affect clinicians’ workplace experiences and how these in turn influence patient outcomes are of interest. A systematic review, currently underway, explores evidence on the association between medical staff gender/ethnicity and resulting patient care. The authors would suggest that the present discussion on sexual harassment and violence in surgery is now broadened to consider the impact (protective or otherwise) of wider factors on medical workforce culture and the effect on staff well-being and patient outcomes.
